# Recent Advances in Understanding of Cardiovascular Diseases in Patients with Chronic Kidney Disease

**DOI:** 10.3390/jcm11164653

**Published:** 2022-08-09

**Authors:** Pajaree Krisanapan, Pattharawin Pattharanitima, Charat Thongprayoon, Wisit Cheungpasitporn

**Affiliations:** 1 Department of Internal Medicine, Division of Nephrology, Faculty of Medicine, Thammasat University, Pathum Thani 12120, Thailand; 2 Division of Nephrology and Hypertension, Department of Medicine, Mayo Clinic, Rochester, MN 55905, USA

Chronic kidney disease (CKD) is a major public health problem, affecting between 8% and 16% of the population worldwide [1,2,3,4,5,6,7]. The global burden of CKD has grown faster than that of other noncommunicable diseases over the previous two decades [8]. This could be explained by a significant increase in disability-adjusted life years (DALYs) as well as a 42–58% increase in CKD-related mortality [9,10]. The leading cause of death among patients with advanced CKD is cardiovascular death, which accounts for half of all deaths [11,12,13,14].

Patients with CKD are known to have increased cardiovascular disease (CVD) complications characterized by coronary artery disease, heart failure, arrhythmias, and sudden cardiac death [12]. In this editorial, “Recent Advances in Understanding of Cardiovascular Diseases in Patients with Chronic Kidney Disease”, we highlight the novelty of recent important studies on the risk factors, prognosis, outcomes, and management of CVD in CKD populations.

CKD is known to be associated with a worse clinical outcome in acute myocardial infarction (AMI) [15,16,17,18]. Utilizing a large inpatient database in the United States, Vallabhajosyula et al., assessed clinical outcomes of AMI with cardiogenic shock (AMI-CS) stratified by different CKD stages. The findings of this study suggested that end-stage kidney disease (ESKD), not CKD, was an independent predictor of greater in-hospital mortality. Interestingly, despite the robustness of guideline-directed therapy, patients with CKD and ESKD were less likely to undergo coronary angiography, percutaneous coronary intervention (PCI), and mechanical circulatory support (MCS) [19]. These findings could possibly be explained by the fact that patients with CKD/ESKD have an increased risk of complications associated with procedures such as contrast-associated acute kidney injury (AKI), as well as thrombotic and bleeding complications [15,17,19,20,21,22]. Nevertheless, future studies are needed to identify strategies to improve the potential health inequities and disparities among patients with CKD/ESKD and CVD.

Atrial fibrillation (AF) is one of the most prevalent arrhythmias in CKD populations, accounting for 15–20% of those with advanced CKD [23,24,25,26,27]. On the other hand, 40–50% of patients with AF also have CKD [26,28,29]. In the *Journal of Clinical Medicine*, Magnocavallo et al., recently reviewed the choice of anticoagulation therapy in patients with AF and CKD [26]. The investigators highlighted the limitations of vitamin K antagonists (VKAs), including a narrow therapeutic window, increased tissue calcification, and low stroke prevention, outweighing major bleeding events. In addition, this review article summarized recent literature on the use of direct oral anticoagulants (DOACs), especially apixaban, that might be superior to VKAs in patients with CKD, including those with ESKD [26].

Patients with CKD are susceptible to not only traditional risk factors for CVD compared with those without CKD but also CKD-specific non-traditional risk factors, including vascular calcification (VC), anemia, sodium and volume retention, and accumulation of uremic toxin, which contribute to worsening atherosclerosis [12,30,31,32,33,34]. While atherosclerosis begins at the endothelium level [35], endothelial dysfunction (ED) in CKD begins early, subsequently progresses with the disease, and significantly leads to cardiovascular complications in these patients [31]. Roumeliotis et al., reviewed the pathophysiology and clinical outcomes of ED in patients with CKD. A hallmark of the development of ED in the CKD population is nitric oxide (NO) reduction, which is caused by multifactorial predisposing factors, including increased endogenous inhibitors of endothelial NO synthase, pro-inflammatory cytokines and oxidative stress, advanced glycosylation products, phosphate, and fibroblast growth factor 23. Additionally, it is affected by a decrease in protective factors such as Klotho and vitamin D. Thus, it is suggested to correct these underlying factors to potentially prevent the progression of atherosclerosis in CKD patients [31].

One of the non-traditional risk factors for developing CVD is VC. KDIGO (Kidney Disease Improving Global Outcomes) states that people with CKD who have VC are at the highest cardiovascular risk due to the fact that VC causes muscular arterial wall thickening and rigidity [36,37,38]. To date, only a lateral abdominal radiograph could be utilized to determine if VC was present or absent [38]. Silaghi et al., comprehensively reviewed the role of serum calciprotein particles (CPPs) and their T_50_, which is the half time needed for spontaneous transition from primary calciprotein particles (CPP I) to secondary calciprotein particles (CPP II), in the diagnosis of developing VC in CKD populations. The findings suggested that T_50_ was shorter in patients with CKD and those with dialysis. Furthermore, a shorter T_50_ was correlated with a higher calcification propensity and was strongly associated with CVD and mortality. As a result, T_50_ may be helpful in the management of VC in patients with CKD, particularly those undergoing hemodialysis [37].

Not only adults but also children with CKD are at increased risk for CVD [39,40,41]. Even though children are less likely to develop overt CVD, atherosclerosis can begin early in life. Thus, it is essential to identify children with CKD at a higher risk for CVD in order to develop an effective prevention strategy [40,42]. Hsu et al. [40] examined the association of gut microbiota-dependent metabolites, including trimethylamine (TMA), trimethylamine N-oxide (TMAO), and dimethylamine (DMA), with cardiovascular risk in children with CKD. This cross-sectional study enrolled 115 children and adolescents with CKD G1-G4 and found that plasma TMA and DMA levels were inversely related to high blood pressure load and estimated glomerular filtration rate (eGFR). Therefore, TMA and DMA are superior to TMAO in terms of cardiovascular risk in children with early stage CKD [40]. Future studies are required to evaluate whether these microbial markers can prognosticate risk for CKD progression in children.

Reduced eGFR is known as a vital independent risk factor for CVD and mortality [12,18,30,33,39]. Thus, the early detection and identification of individuals at high risk for CKD progression are crucial for optimizing patient management. Low high-density lipoprotein-cholesterol (HDL-c) is one of the most significant lipid abnormalities in patients with mild to moderate CKD and is associated with reduced lecithin cholesterol acyltransferase (LCAT) concentration [43,44]. A recent investigation by Baragetti et al. found that reduced circulating LCAT levels predicted CKD progression at the early stages of renal dysfunction independent of changes in HDL-c levels. Thus, it is hypothesized that pharmacologic therapy that targets LCAT and restores HDL-c could decrease the development of CVD in those with CKD [44].

Recently, Yu et al. [45] applied unsupervised machine learning and hierarchical clustering with heatmap visualization to classify CKD staging and to predict the progression of CKD. This retrospective cohort study found that obesity, hyperglycemia, and liver function were highly associated with CKD. Interestingly, hypertension and HbA1c were in the same cluster with a similar pattern, whereas HDL-c had the opposite pattern because higher HDL-c indicates a healthy state. The application of machine learning approaches may aid physicians in making management decisions for patients in the CKD high-risk group [45].

In conclusion, recent findings published in the *Journal of Clinical Medicine* have provided more understanding and additional knowledge that may help physicians improve the management and outcomes of CVD in patients with CKD.
